# Clinical Impact of Semaglutide in Patients with Heart Failure and Preserved Ejection Fraction

**DOI:** 10.3390/jcm15124831

**Published:** 2026-06-22

**Authors:** Yuki Hida, Teruhiko Imamura, Koichiro Kinugawa

**Affiliations:** The Second Department of Internal Medicine, University of Toyama, 2630 Sugitani, Toyama 930-0194, Japan; time.to.control5@gmail.com (Y.H.);

**Keywords:** semaglutide, heart failure with preserved ejection fraction, B-type natriuretic peptide, type 2 diabetes mellitus, C-reactive protein, cardiac remodeling

## Abstract

**Background**: The clinical impact of oral semaglutide on cardiac biomarkers in real-world patients with heart failure with preserved ejection fraction (HFpEF) and type 2 diabetes mellitus (T2DM) remains unclear. We evaluated whether initiation of oral semaglutide was associated with a reduction in B-type natriuretic peptide (BNP) levels and explored factors associated with this response. **Methods:** We retrospectively enrolled 27 patients with HFpEF who initiated oral semaglutide for T2DM management at a single academic center. Clinical data were collected at three time points: three months before treatment initiation (pre-treatment period), at initiation (baseline), and three months after semaglutide initiation (on-treatment period). The primary outcome was the change in the common logarithm of BNP levels (log BNP) during the on-treatment versus pre-treatment period. **Results:** Median age was 67 (59, 78) years and 21 (77.8%) were men. Log BNP remained stable during the pre-treatment period (*p* = 0.34) but decreased significantly during the on-treatment period (*p* < 0.001). The reduction in log BNP during the on-treatment period was significantly greater than during the pre-treatment period (mean difference −0.35, 95% confidence interval −0.44 to −0.11, *p* < 0.001). Concomitant reductions were observed in HbA1c, body weight, C-reactive protein, left atrial volume index, and left ventricular mass index. Changes in C-reactive protein levels were significantly correlated with those in log BNP (r = 0.46, *p* = 0.015). **Conclusions:** In patients with HFpEF and T2DM, three-month oral semaglutide therapy was associated with reductions in BNP, as well as improvements in glycemic control, systemic inflammation, left atrial volume index, and left ventricular mass index.

## 1. Introduction

Heart failure with preserved ejection fraction (HFpEF) is an increasingly recognized public health challenge, accounting for more than half of all heart failure cases and carrying substantial morbidity and mortality [1,2,3]. Unlike heart failure with reduced ejection fraction, HFpEF lacks well-established therapies with proven mortality benefit, often driven by cardiometabolic comorbidities including obesity, type 2 diabetes mellitus (T2DM), hypertension, and systemic inflammation [4,5,6]. These comorbidities contribute to myocardial remodeling, increased cardiac filling pressures, and progressive functional impairment, contributing to the increasing prevalence of HFpEF worldwide.

Glucagon-like peptide-1 receptor agonists (GLP-1RAs) were initially developed to improve glycemic control in T2DM but have since shown a wide range of cardiometabolic benefits extending beyond blood glucose reduction [7]. Semaglutide, a potent GLP-1RA available in both subcutaneous and oral formulations, has been shown to reduce body weight, improve glycemic control, and lower the risk of major adverse cardiovascular events [8,9,10]. Large-scale clinical trials using high-dose subcutaneous semaglutide have demonstrated favorable cardiovascular and metabolic effects. In the SELECT trial, weekly subcutaneous semaglutide 2.4 mg lowered the incidence of major adverse cardiovascular events among patients with overweight or obesity and pre-existing cardiovascular disease [11,12]. The STEP-HFpEF program demonstrated that high-dose subcutaneous semaglutide significantly improved quality of life, exercise capacity, and body weight in obese patients with HFpEF and reduced natriuretic peptides and C-reactive protein (CRP), suggesting favorable effects on cardiac congestion and systemic inflammation [13,14,15].

However, current evidence is largely derived from trials using high-dose subcutaneous formulations administered for obesity management. In contrast, oral semaglutide is administered at standard diabetes doses and may offer practical advantages over subcutaneous formulations, including greater convenience and potentially improved treatment acceptance in routine clinical practice. Nevertheless, the clinical impact of oral semaglutide in real-world patients with HFpEF and T2DM remains unclear, particularly regarding changes in B-type natriuretic peptide (BNP). Therefore, we retrospectively evaluated the clinical impact of oral semaglutide in patients with HFpEF and T2DM. 

## 2. Methods

### 2.1. Study Design and Patient Selection

This was a retrospective observational study conducted at a large academic institute. We consecutively enrolled patients with HFpEF who initiated oral semaglutide therapy between 2022 and 2025.

HFpEF was defined as a left ventricular ejection fraction ≥ 50% in the presence of clinical signs and/or symptoms of heart failure, according to the Framingham criteria. Patients with available clinical data at three time points—three months before the initiation of semaglutide, at the time of initiation (baseline), and three months after initiation—were included. The three-month duration before the initiation of semaglutide was defined as a pre-treatment period, and the three-month duration during semaglutide therapy was defined as an on-treatment period. Patients with missing data at these time points were excluded.

The study protocol was approved by the institutional review board of the Ethics Committee, University of Toyama (approval number: R2015154), and the study was conducted in accordance with the Declaration of Helsinki. The requirement for informed consent was waived due to the retrospective nature of the study.

### 2.2. Semaglutide Therapy

Oral semaglutide was prescribed for the management of T2DM in accordance with current clinical practice guidelines [16]. In general, GLP-1RAs are indicated for patients with inadequate glycemic control despite lifestyle modification and/or other antidiabetic medications and are particularly considered in those with overweight or obesity or increased cardiovascular risk.

In the present study, the initiation of semaglutide therapy was determined by the treating physicians based on clinical judgment, taking into account glycemic control, body weight, and overall cardiovascular risk profile.

Semaglutide was administered as an oral formulation according to standard clinical practice. Treatment was initiated at a dose of 3 mg once daily, followed by escalation to 7 mg and, when appropriate, to 14 mg at intervals of approximately 4 weeks, based on tolerability and glycemic response.

Dose adjustments were made at the discretion of the treating physicians, taking into account gastrointestinal tolerability, glycemic control, and overall clinical status. Concomitant antidiabetic and heart failure medications were continued or adjusted as clinically indicated.

Patients were routinely monitored during the treatment period, including assessment of symptoms, body weight, and laboratory parameters. No predefined protocol for medication adjustment was applied, reflecting real-world clinical practice.

### 2.3. Data Collection

Clinical data were collected from electronic medical records. Demographic data, comorbidities, laboratory findings, echocardiographic parameters, and medication use were obtained at each time point. BNP values were analyzed as the common logarithm (log BNP) due to their skewed distribution. Left ventricular ejection fraction was calculated by the modified Simpson’s method. Left atrial volume and left ventricular mass were indexed by body surface area.

### 2.4. Study Endpoints

The primary outcome was the change in log BNP during the on-treatment period versus during the pre-treatment period. The changes in other clinical parameters were also assessed as secondary outcomes. 

### 2.5. Statistical Analysis

Continuous variables are presented as median (interquartile range), and categorical variables as number (percentage). A two-sided *p*-value < 0.05 was considered statistically significant. All statistical analyses were performed using SPSS Statistics 23 (SPSS Inc., Armonk, IL, USA).

Changes in clinical continuous variables across the three time points were assessed using the Friedman test for repeated measures. Pairwise comparisons were performed using the Wilcoxon signed-rank test. Changes in clinical categorical variables across the three time points were assessed using Cochran’s Q test.

Changes in log BNP during the pre-treatment and on-treatment periods were compared using paired analyses, and the difference in changes between the two periods was evaluated. The effect size and corresponding 95% confidence intervals were estimated using bootstrap resampling.

To evaluate the trajectory of BNP over time, a piecewise linear mixed-effects model was constructed, with separate slopes for the pre-treatment and on-treatment periods and a random intercept for each patient.

Sensitivity analyses were performed by stratifying patients according to baseline body mass index (>25 kg/m^2^), left ventricular mass index (>90 g/m^2^), and baseline log BNP (>2.35) and comparing changes in log BNP between groups using the Mann–Whitney U test. Associations between changes in BNP and changes in other clinical variables were assessed using Pearson’s correlation coefficient. 

To identify independent predictors of BNP changes, multivariable linear regression analysis was performed, including baseline log BNP, estimated glomerular filtration rate, and body mass index as covariates, considering their impacts on clinical outcomes in the heart failure patients.

## 3. Results

### 3.1. Baseline Characteristics

A total of 27 patients who received semaglutide therapy were included in this retrospective study (Table 1). The median age was 67 (59, 78) years, and 21 patients (77.8%) were men. The median body mass index was 26.2 (22.9, 27.5) kg/m^2^, and the median HbA1c (NGSP) level was 8.0% (7.2, 8.8). The median log BNP was 2.35 (2.19, 2.43) pg/mL. All patients had HFpEF, with a median left ventricular ejection fraction of 56% (52, 62).

Renal function was moderately impaired, with a median estimated glomerular filtration rate of 51.4 (36.1, 60.7) mL/min/1.73 m^2^. Inflammatory status was relatively mild, with a median CRP level of 0.24 (0.13, 0.71) mg/dL.

Most patients were receiving guideline-directed medical therapy for heart failure, including beta-blockers (96.3%), renin–angiotensin system inhibitors (88.9%), mineralocorticoid receptor antagonists (51.9%), and sodium–glucose cotransporter 2 inhibitors (77.8%).

### 3.2. Feasibility and Safety of Semaglutide Therapy

During the 3-month treatment period, semaglutide therapy was generally well tolerated. No drug-related adverse events were observed, and all patients continued the therapy throughout the observation period. One patient required hospitalization due to worsening heart failure; however, a direct causal relationship with semaglutide could not be established. No deaths occurred during the study period.

The administration of other diabetic medications and heart failure medications remained unchanged both during the pre-treatment and on-treatment periods (all *p* > 0.05). 

### 3.3. Trajectory of BNP Levels

**Figure 1:**
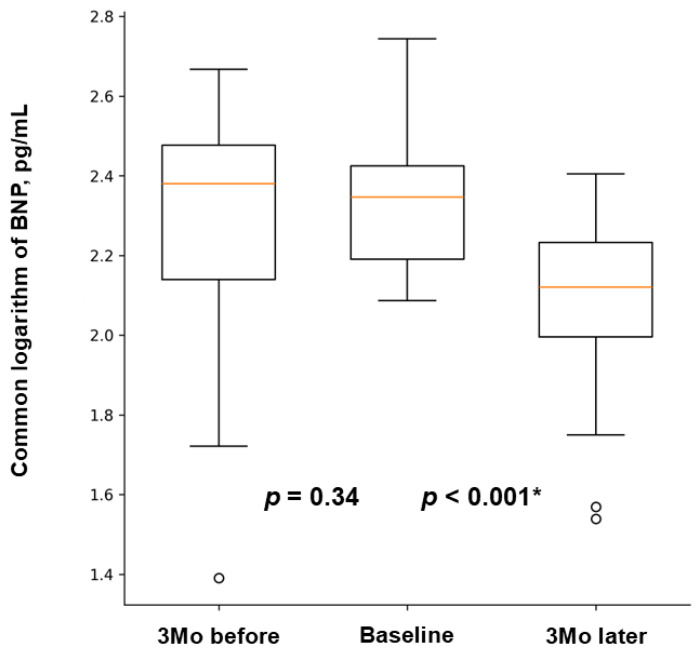
Trajectory of BNP levels before and after the initiation of semaglutide. Common logarithm of BNP levels were followed three months before, at baseline, and three months after the initiation of semaglutide. BNP levels were compared by the Friedman test and post hoc Wilcoxon signed-rank test. BNP level remained unchanged before the initiation of semaglutide, whereas BNP levels decreased significantly during three-month semaglutide therapy. * *p* < 0.05. BNP, B-type natriuretic peptide.

The log BNP remained unchanged during the pre-treatment period (2.38 [2.14–2.48] to 2.35 [2.19–2.43], *p* = 0.34), whereas it decreased significantly during the on-treatment period to 2.12 (2.00–2.23) (*p* < 0.001) (Figure 1).

**Figure 2:**
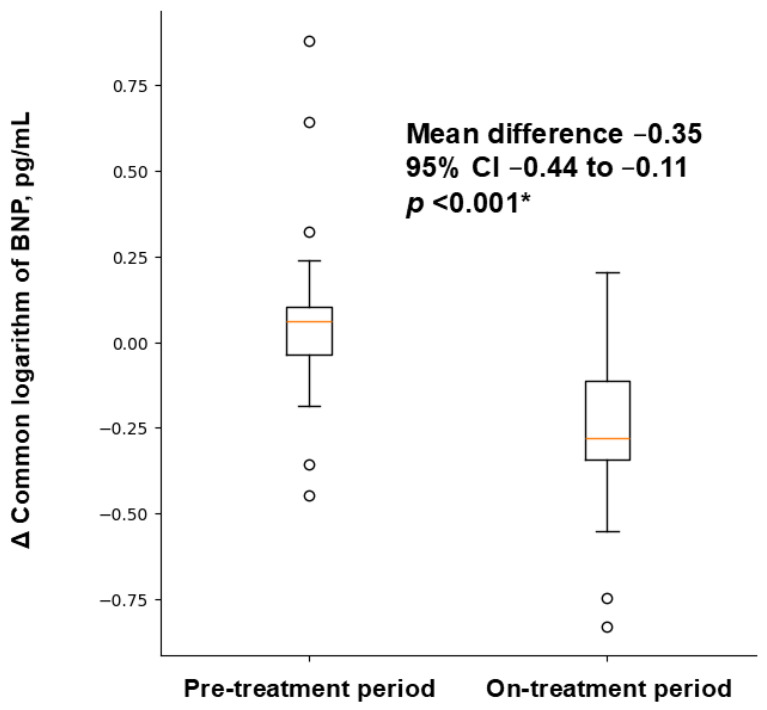
Changes in BNP levels during the pre-treatment period versus the on-treatment period. Change in BNP levels was approximately zero during the pre-treatment period, whereas the change in BNP levels was significantly lower during the on-treatment period than the pre-treatment period. * *p* < 0.05 by the Mann–Whitney U test. BNP, B-type natriuretic peptide; CI, confidence interval.

Consistently, the median change in log BNP was minimal during the pre-treatment period (0.06 [−0.037 to 0.103]) but showed a marked reduction during the on-treatment period (−0.281 [−0.341 to −0.113]), with a significant difference between the two periods (*p* < 0.001) (Figure 2). The reduction in log BNP after the initiation of semaglutide was significantly greater than that observed during the pre-treatment period (mean difference −0.35, 95% confidence interval −0.44 to −0.11).

In a piecewise linear mixed-effects model, the slope during the pre-treatment period was not significant (β = 0.071, 95% confidence interval −0.026 to 0.169, *p* = 0.153), whereas a significant decline was observed during the on-treatment period (β = −0.262, 95% confidence interval −0.360 to −0.164, *p* < 0.001). The difference between the two slopes was also significant (β = −0.333, 95% confidence interval −0.502 to −0.164, *p* < 0.001).

### 3.4. Trajectory of Other Clinical Variables

The trajectories of other clinical variables are summarized in Table 2. HbA1c (NGSP) remained unchanged during the pre-treatment period but decreased significantly at three months after the initiation of semaglutide (*p* < 0.001).

Similarly, body weight, serum CRP, left atrial volume index, and left ventricular mass index showed significant reductions after treatment initiation, whereas no consistent changes were observed during the pre-treatment period.

### 3.5. Sensitivity Analyses

**Figure 3:**
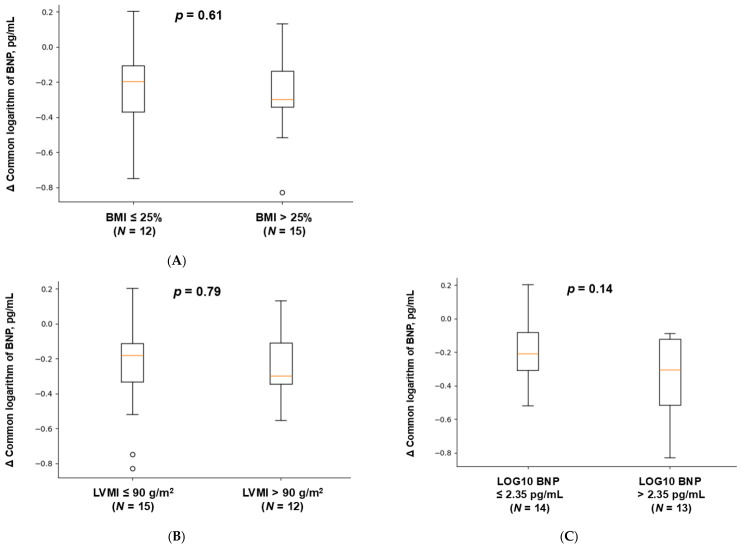
Sensitivity analyses for the changes in BNP levels. (**A**) BMI, (**B**) LVMI, and (**C**) common logarithm of BNP. Patients were divided by the cutoffs of each value. The reduction of BNP was consistent without any interactions between the two groups, which was compared by the Mann–Whitney U test. BMI, body mass index; LVMI, left ventricular mass index; BNP, B-type natriuretic peptide.

The cohort was stratified according to baseline body mass index, left ventricular mass index, and the log BNP (Figure 3A–C). The reduction in log BNP during the on-treatment period did not differ significantly between groups stratified by body mass index or left ventricular mass index (*p* = 0.61 and *p* = 0.79, respectively).

When stratified by baseline log BNP (cut-off: 2.35), patients with higher baseline values tended to show greater reductions in log BNP; however, this difference did not reach statistical significance (*p* = 0.14).

### 3.6. Impact of Baseline Variables on the Changes in BNP

In multivariable linear regression analysis adjusted for baseline log BNP, estimated glomerular filtration rate, and body mass index, only baseline log BNP was independently associated with subsequent changes in log BNP. Higher baseline log BNP was significantly associated with a greater reduction in log BNP levels (β = −0.65, 95% confidence interval −1.15 to −0.14, *p* = 0.014) (Table 3), whereas baseline estimated glomerular filtration rate and body mass index were not significantly associated with log BNP changes.

### 3.7. Association Between Changes in Clinical Variables and BNP

**Figure 4:**
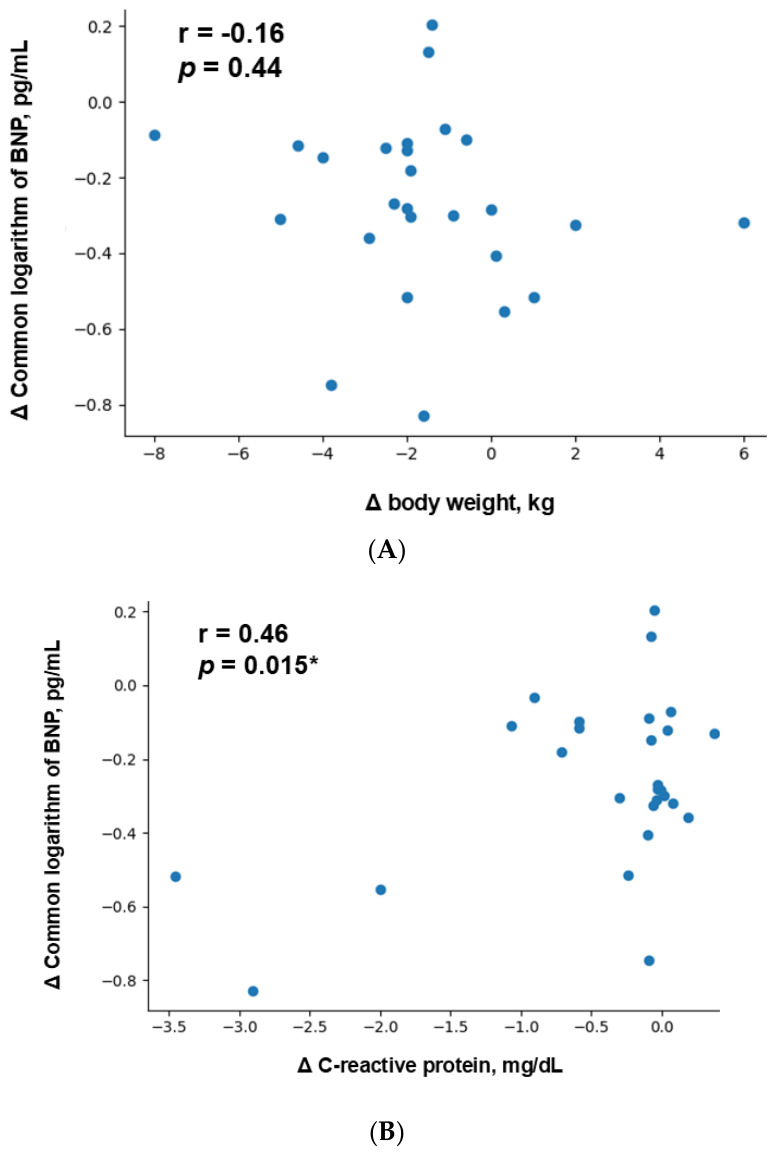
Correlation between the changes in BNP and those of clinical variables. (**A**) Body weight and (**B**) CRP. Change in BNP was not significantly correlated with those in body weight but was significantly correlated with those in serum CRP level. BNP, B-type natriuretic peptide; CRP, C-reactive protein. * *p* < 0.05 by Pearson’s correlation coefficient.

Changes in body weight were not significantly correlated with changes in log BNP (r = −0.16, *p* = 0.44). In contrast, changes in CRP showed a modest but significant positive correlation with changes in log BNP (r = 0.46, *p* = 0.015) (Figure 4A,B).

## 4. Discussion

### 4.1. Principal Findings

The present study demonstrated that the initiation of oral semaglutide was associated with a significant reduction in BNP levels in real-world patients with HFpEF and T2DM, whereas BNP remained stable during the pre-treatment period without oral semaglutide under otherwise unchanged medical management. This pre–post design with a confirmed stable medication background throughout both observation periods argues against spontaneous fluctuation or change in background therapy as explanations for the observed BNP reduction. In addition to BNP, significant improvements were observed in HbA1c, body weight, serum CRP, left atrial volume index, and left ventricular mass index, reflecting cardiometabolic improvement across multiple domains. Oral semaglutide therapy was well tolerated, with no drug-related adverse events and no treatment discontinuations during the observation period.

### 4.2. Conceptual Role of Oral Semaglutide in HFpEF

HFpEF is a heterogeneous syndrome including several distinct phenotypes, including hypertensive, atrial fibrillation-related, elderly frail, and obese or cardiometabolic phenotypes [17,18]. Among these, the cardiometabolic phenotype has emerged as a major phenotype, particularly among younger patients with T2DM, excess adiposity, and insulin resistance [17]. A central pathophysiological feature of this phenotype is the prominence of chronic systemic inflammation, driven by adipose tissue-derived pro-inflammatory cytokines, adipokine dysregulation, and oxidative stress [19]. Compared with other HFpEF phenotypes, the cardiometabolic phenotype is characterized by higher circulating levels of inflammatory markers, such as CRP and interleukin-6, which promote microvascular endothelial dysfunction, myocardial fibrosis, and impaired diastolic relaxation [4,20,21]. The present cohort, consisting of patients with T2DM and overweight or mild obesity, closely reflects this phenotype. 

In contrast to conventional heart failure therapies such as renin-angiotensin system inhibitors, mineralocorticoid receptor antagonists, or sodium-glucose cotransporter 2 inhibitors [22,23,24]—which act primarily through neurohormonal or renal mechanisms—GLP-1RAs offer a different profile of pleiotropic effects including adiposity reduction, glycemic improvement, blood pressure lowering, and anti-inflammation action [25,26]. These properties may be particularly relevant in the cardiometabolic HFpEF phenotype [25]. It should be noted that the STEP-HFpEF program used high-dose subcutaneous semaglutide for obesity management, whereas the present study employed oral semaglutide at standards diabetes doses; the cardiovascular effects of these two formulations and regimens may not be directly comparable. Our findings suggest that oral semaglutide, even at standard diabetes doses, may improve cardiac biomarkers and cardiac structure in real-world patients with cardiometabolic HFpEF.

### 4.3. Impact of Semaglutide on Inflammatory Status

An important observation in the present study was that changes in CRP were significantly correlated with changes in log BNP, whereas changes in body weight showed no significant correlation. This finding suggests that anti-inflammatory effects, rather than weight loss alone, may be related to the cardiac benefits of semaglutide. 

GLP-1RAs have been shown to reduce systemic inflammation through several pathways, including downregulation of pro-inflammatory cytokines such as interleukin-6 and tumor necrosis factor-α, reduction of oxidative stress, and shifts in macrophage polarization toward an anti-inflammatory phenotype [27,28]. Chronic systemic inflammation is a cardinal pathophysiological feature of HFpEF, driving microvascular endothelial dysfunction, myocardial fibrosis, and impaired diastolic function. Attenuation of this inflammatory state may therefore contribute to reduced myocardial wall stress and lower natriuretic peptide levels [5,6]. Consistent with this hypothesis, the STEP-HFpEF trial demonstrated a 43.5% reduction in CRP with semaglutide [25]. The mechanistic implication of the CRP-BNP correlation identified in the present study is further supported by the broader context of unresolved inflammatory drivers in chronic HF progression [29]. Of note, epicardial adipose tissue (EAT)—a metabolically active fat depot located between the myocardium and the visceral pericardium that secretes pro-inflammatory adipokines with direct paracrine effects on the myocardium—was not assessed in the present study. Given that semaglutide may preferentially reduce ectopic fat depots including EAT, future studies incorporating EAT measurement would help to clarify the inflammatory mechanisms behind the BNP response observed here [30].

### 4.4. Impact of Semaglutide on Body Weight

The dissociation between weight change and BNP reduction in the present study merits further consideration. Although body weight decreased significantly after semaglutide initiation, the magnitude of weight reduction was modest compared with that reported in the STEP-HFpEF program. Previous studies suggest that semaglutide reduces body weight across a broad range of baseline body mass index [11,12,31]. The relatively limited weight reduction in the present cohort, probably reflecting the lower baseline body mass index and use of standard diabetes doses, may explain the absence of a correlation between weight change and BNP reduction.

### 4.5. Future Perspectives

The present findings suggest several future research directions. Large-scale prospective randomized controlled trials are warranted to confirm the cardiometabolic benefits of oral semaglutide in patients with HFpEF and T2DM. Future studies should incorporate longer follow-up periods to assess the durability of BNP reduction and the impact on clinical endpoints, including heart failure hospitalization, mortality, and patient-reported outcomes such as Kansas City Cardiomyopathy Questionnaire (KCCQ) and six-minute walk test (6MWT). Additionally, assessment of epicardial adipose tissue and other inflammatory biomarkers would help clarify the mechanisms behind the cardiac effects of semaglutide in this population.

### 4.6. Limitations

This study has several limitations. The retrospective single-center design without a randomized control group limits causal inference. Although the pre-treatment period served as an internal control and concomitant medications were confirmed to be unchanged, unmeasured confounders may have influenced the results. Furthermore, the high prevalence of concomitant SGLT2 inhibitor use (77.8%) may limit the attribution of BNP reduction solely to semaglutide, although medications including SGLT2 inhibitors remained unchanged throughout both periods (Table 2). A formal a priori power calculation was not performed, and the small sample size of 27 patients limits the statistical power of subgroup and correlation analyses; accordingly, the findings should be interpreted as exploratory rather than confirmatory. Patient-reported outcomes and functional capacity, including health-related quality of life assessed by the KCCQ and exercise tolerance evaluated by the 6MWT, were not systematically assessed, which further limits the clinical interpretation of the present findings. The three-month follow-up precludes assessment of longer-term effects on clinical events such as heart failure hospitalization and mortality. This study is therefore considered exploratory, and prospective, longer-term investigation is warranted to confirm the durability of these findings. The present cohort predominantly reflects the obese or cardiometabolic HFpEF phenotype, characterized by T2DM, adiposity-related inflammation, and metabolic dysregulation. HFpEF is a heterogeneous syndrome that includes distinct phenotypes—including hypertensive, atrial fibrillation-related, and elderly frail phenotypes—for which the efficacy of semaglutide may differ. Accordingly, the generalizability of the present findings to non-cardiometabolic HFpEF phenotypes remains uncertain. Furthermore, HFpEF diagnosis in the present study was based on LVEF ≥ 50% combined with Framingham criteria, without systematic application of contemporary scoring systems such as the HFA-PEFF algorithm or H2FPEF score; this may limit the diagnostic rigor and comparability with future studies employing current diagnostic standards. BNP, rather than NT-pro BNP, was used as the primary natriuretic peptide marker, reflecting institutional practice in Japan, where BNP is the predominant biomarker used in clinical guidance. The absence of NT-pro BNP data may limit direct comparability with international HFpEF trials.

## 5. Conclusions

In this real-world retrospective study, the initiation of oral semaglutide was associated with a reduction in BNP levels, along with improvements in glycemic control, body weight, systemic inflammation, left atrial volume index, and left ventricular mass index in patients with HFpEF and T2DM. Change in CRP was closely associated with BNP improvement, suggesting that anti-inflammatory mechanisms may be involved in the observed cardiac effects of semaglutide in this population. These findings suggest the potential cardiometabolic relevance of oral semaglutide in HFpEF patients with T2DM.

## Figures and Tables

**Table 1:** Baseline characteristics.

	*N* = 27
Demographics	
Age, years	67 (59, 78)
Men	21 (77.8%)
Body height, cm	162.0 (158.5, 168.0)
Body weight, kg	69.0 (60.3, 72.3)
Body mass index	26.2 (22.9, 27.5)
Body surface area, m^2^	1.77 (1.61, 1.82)
Comorbidity	
Hypertension	20 (74.1%)
Dyslipidemia	16 (59.3%)
Atrial fibrillation	13 (48.1%)
Coronary artery disease	4 (14.8%)
Diabetes mellitus	27 (100%)
Laboratory data	
Hemoglobin, g/dL	14.7 (12.8, 16.1)
Serum albumin, g/dL	3.9 (3.8, 4.1)
Serum sodium, mEq/L	138 (138, 140)
Serum potassium, mEq/L	4.5 (4.2, 4.7)
Serum total bilirubin, mg/dL	1.0 (0.6, 1.1)
Estimated glomerular filtration rate, mL/min/1.73 m^2^	51.4 (36.1, 60.7)
Common logarithm of B-type natriuretic peptide, pg/mL	2.35 (2.19, 2.43)
HbA1c (NGSP), %	8.0 (7.2, 8.8)
Serum C-reactive protein, mg/dL	0.24 (0.13, 0.71)
Serum triglyceride, mg/dL	152 (100, 254)
Echocardiography	
Left ventricular end-diastolic diameter, mm	49 (46, 54)
Left ventricular ejection fraction, %	56 (52, 62)
Left atrial volume index, mL/m^2^	34.0 (27.5, 58.1)
Interventricular septum thickness, mm	9 (8, 10)
Posterior wall thickness, mm	9 (9, 10)
Left ventricular mass index, g/m^2^	87.9 (78.3, 99.5)
Diabetic medications	
Dipeptidyl peptidase-4 inhibitors	14 (51.9%)
Metformin	10 (37.0%)
Imeglimin hydrochloride	1 (3.7%)
Insulin	1 (3.7%)
Heart failure medications	
Beta-blockers	26 (96.3%)
Renin-angiotensin system inhibitors	24 (88.9%)
Mineralocorticoid receptor antagonists	14 (51.9%)
Sodium-glucose cotransporter 2 inhibitors	21 (77.8%)

Clinical data were obtained just before the initiation of semaglutide therapy. Continuous variables are stated as median (25% interquartile, 75% interquartile), and categorical variables are stated as numbers (percentages).

**Table 2:** Trajectory of clinical data.

	Three Months Before	Baseline	Three Months Later	*p*-Value
Demographics				
Body weight, kg	67.7 (58.5, 74.5)	69.0 (60.3, 72.3)	67.0 (57.4, 70.9)	<0.001 *
Body mass index	26.1 (22.7, 27.9)	26.2 (22.9, 27.5)	25.2 (22.2, 26.9)	<0.001 *
Laboratory data				
Hemoglobin, g/dL	14.2 (12.4, 16.4)	14.7 (12.8, 16.1)	14.1 (12.4, 16.5)	0.38
Serum albumin, g/dL	3.8 (3.6, 4.1)	3.9 (3.8, 4.1)	4.0 (3.9, 4.2)	0.24
Serum sodium, mEq/L	140 (138, 141)	138 (138, 140)	139 (138, 141)	0.18
Serum potassium, mEq/L	4.6 (4.3, 4.7)	4.5 (4.2, 4.7)	4.5 (4.2, 4.8)	0.14
Serum total bilirubin, mg/dL	0.7 (0.5, 0.9)	1.0 (0.6, 1.1)	0.7 (0.6, 0.9)	0.003 *
eGFR, mL/min/1.73 m^2^	48.8 (37.7, 60.5)	51.4 (36.1, 60.7)	50.2 (36.0, 63.5)	0.77
HbA1c (NGSP), %	7.6 (7.0, 8.0)	8.0 (7.2, 8.8)	7.5 (6.5, 8.0)	<0.001 *
Serum C-reactive protein, mg/dL	0.17 (0.08, 0.32)	0.24 (0.13, 0.71)	0.09 (0.06, 0.21)	0.002 *
Serum triglyceride, mg/dL	161 (98, 213)	152 (100, 254)	133 (100, 196)	0.097
Echocardiography data				
Left ventricular end-diastolic diameter, mm	49 (45, 52)	49 (46, 54)	48 (46, 52)	0.11
Left ventricular ejection fraction, %	58 (51, 64)	56 (52, 62)	58 (51, 64)	0.96
Left atrial volume index, mL/m^2^	33.1 (25.5, 55.4)	34.0 (27.5, 58.1)	31.5 (24.6, 47.0)	0.007 *
Interventricular septum thickness, mm	9 (8, 10)	9 (8, 10)	9 (8, 9)	0.009 *
Posterior wall thickness, mm	9 (8, 10)	9 (9, 10)	9 (8, 10)	0.004 *
Left ventricular mass index, g/m^2^	89.5 (74.6, 94.2)	87.9 (78.3, 99.5)	80.4 (66.9, 91.9)	0.004 *
Diabetic medications				
Dipeptidyl peptidase-4 inhibitors	18 (66.7%)	14 (51.9%)	14 (51.9%)	0.76
Metformin	9 (33.3%)	10 (37.0%)	11 (40.7%)	0.22
Imeglimin hydrochloride	1 (3.7%)	1 (3.7%)	1 (3.7%)	-
Insulin	1 (3.7%)	1 (3.7%)	1 (3.7%)	-
Heart failure medications				
Beta-blockers	26 (96.3%)	26 (96.3%)	27 (100.0%)	0.61
Renin-angiotensin system inhibitors	23 (85.2%)	24 (88.9%)	25 (92.6%)	0.22
Mineralocorticoid receptor antagonists	14 (51.9%)	14 (51.9%)	14 (51.9%)	-
Sodium-glucose cotransporter 2 inhibitors	21 (77.8%)	21 (77.8%)	22 (81.5%)	0.37

Clinical data obtained at the three points: three months before the initiation of semaglutide, baseline, and three months after the initiation of semaglutide. Continuous variables are stated as median (25% interquartile, 75% interquartile), and categorical variables are stated as numbers (percentages). The trend was assessed by the Friedman test or the McNemar test. * *p* < 0.05. eGFR, estimated glomerular filtration rate.

**Table 3:** Baseline variables and changes in BNP.

	Beta-Value	95% CI	*p*-Value
Baseline common logarithm of BNP, pg/mL	−0.65	−1.15 to −0.14	0.014 *
Baseline eGFR, mL/min/1.73 m^2^	0.002	−0.003 to 0.007	0.39
Baseline body mass index	−0.005	−0.022 to 0.012	0.58

Multivariable linear regression analysis was performed to evaluate the impact of pre-defined baseline variables on the changes in BNP. BNP, B-type natriuretic peptide; eGFR, estimated glomerular filtration rate; CI, confidence interval. * *p* < 0.05.

## Data Availability

Data are available from the corresponding author upon reasonable request.
